# Atypical intrinsic neural timescale in the left angular gyrus in Alzheimer’s disease

**DOI:** 10.1093/braincomms/fcae199

**Published:** 2024-07-11

**Authors:** Shota A Murai, Tatsuo Mano, Jerome N Sanes, Takamitsu Watanabe

**Affiliations:** International Research Center for Neurointelligence (WPI-IRCN), The University of Tokyo Institutes for Advanced Study, Bunkyo City, Tokyo 113-0033, Japan; Department of Degenerative Neurological Diseases, National Center of Neurology and Psychiatry, Tokyo 187-8551, Japan; Department of Neuroscience, Brown University, Providence, RI 02912, USA; Carney Institute for Brain Science, Brown University, Providence, RI 02912, USA; Center for Neurorestoration and Neurotechnology, Veterans Affairs Providence Healthcare System, Providence, RI 02908, USA; International Research Center for Neurointelligence (WPI-IRCN), The University of Tokyo Institutes for Advanced Study, Bunkyo City, Tokyo 113-0033, Japan

**Keywords:** Alzheimer’s disease, intrinsic neural timescale, left angular gyrus, default mode network, grey matter volume

## Abstract

Alzheimer’s disease is characterized by cognitive impairment and progressive brain atrophy. Recent human neuroimaging studies reported atypical anatomical and functional changes in some regions in the default mode network in patients with Alzheimer’s disease, but which brain area of the default mode network is the key region whose atrophy disturbs the entire network activity and consequently contributes to the symptoms of the disease remains unidentified. Here, in this case–control study, we aimed to identify crucial neural regions that mediated the phenotype of Alzheimer’s disease, and as such, we examined the intrinsic neural timescales—a functional metric to evaluate the capacity to integrate diverse neural information—and grey matter volume of the regions in the default mode network using resting-state functional MRI images and structural MRI data obtained from individuals with Alzheimer’s disease and cognitively typical people. After confirming the atypically short neural timescale of the entire default mode network in Alzheimer’s disease and its link with the symptoms of the disease, we found that the shortened neural timescale of the default mode network was associated with the aberrantly short neural timescale of the left angular gyrus. Moreover, we revealed that the shortened neural timescale of the angular gyrus was correlated with the atypically reduced grey matter volume of this parietal region. Furthermore, we identified an association between the neural structure, brain function and symptoms and proposed a model in which the reduced grey matter volume of the left angular gyrus shortened the intrinsic neural time of the region, which then destabilized the entire neural timescale of the default mode network and resultantly contributed to cognitive decline in Alzheimer’s disease. These findings highlight the key role of the left angular gyrus in the anatomical and functional aetiology of Alzheimer’s disease.

## Teaser

By scrutinizing local neural timescales in the brains of patients with Alzheimer’s disease, Murai *et al*. have found that atrophy of the parietal region destabilizes the area’s activity, which triggers the unstable activity of the entire default mode network and results in the symptoms of Alzheimer’s disease.

## Graphical Abstract

**Graphical Abstract:**
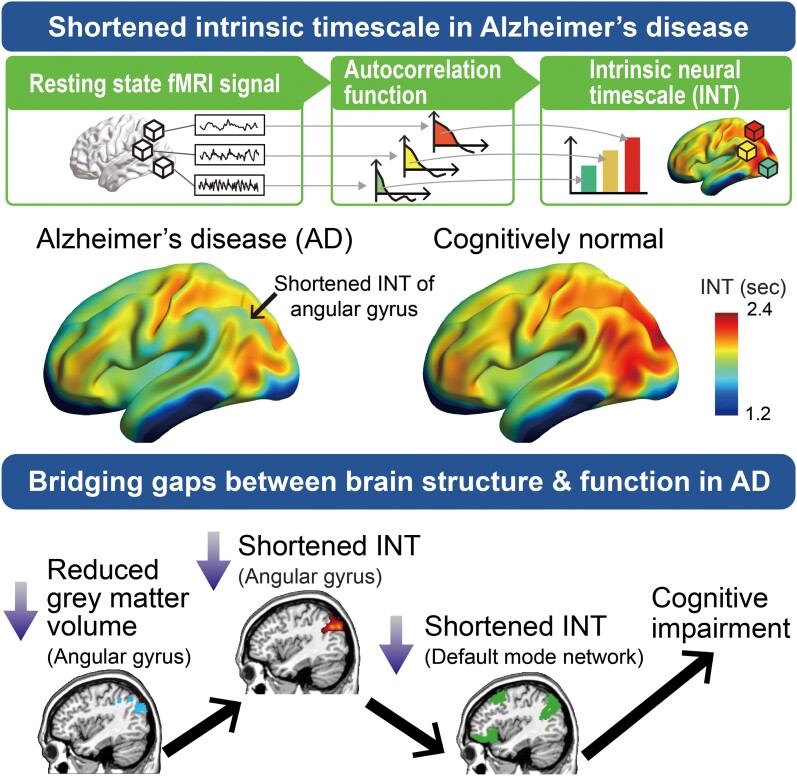

## Introduction

Alzheimer’s disease is the most common form of dementia, ostensibly due to progressive brain atrophy. In both humans and non-human animal model systems, pathological findings show that cognitive impairment in Alzheimer’s disease and its neural degeneration are specifically associated with amyloid and tau depositions in myriad brain regions, including the medial and lateral temporal lobes and several parietal regions.^1-7^

Neural degenerative changes in Alzheimer’s disease commonly occur in regions within the so-called default mode network (DMN),^8,9^ including the medial prefrontal cortex, precuneus and angular gyrus,^1,10-15^ and the atrophy seen in some of these DMN regions is specific to Alzheimer’s disease.^12-14^

Moreover, Alzheimer’s disease yields atypical intrinsic activity in the DMN as assessed by resting-state functional magnetic resonance imaging (rsfMRI).^16,17^ Specifically, Alzheimer’s disease patients showed significantly weaker functional connectivity within the entire DMN^16,18^ or only its posterior part,^19,20^ and such atypical functional connectivity in the DMN was associated with the deposition of amyloid^1,20,21^ and tau^22-24^ in the network. Given the DMN’s crucial role in cognitive capability in older adults,^25^ these findings led us to hypothesize that the anatomical changes within the DMN would disturb its neural dynamics, which then might become causally linked to the cognitive impairment of Alzheimer’s disease.

However, it remains unclear whether one or more brain regions in the DMN have such a critical role in the pathophysiology of Alzheimer’s disease. Anatomically, multiple neuroimaging studies described Alzheimer’s disease-specific atrophy in the temporal-parietal junction,^12-14^ but whether such focal atrophy in the DMN affects the network’s neural activity in Alzheimer’s disease was not examined. Several functional MRI studies with Alzheimer’s disease patients identified atypical neural dynamics in several parietal regions within the DMN;^19,20,26^ but these studies did not directly test whether such aberrant neural activation was attributable to local brain atrophy. Consequently, the DMN region whose focal anatomical change induces network-level neural dysfunction that leads to Alzheimer’s disease symptoms in humans has been unidentified.

Here, we examined the ‘intrinsic neural timescale’ (INT) of resting-state neural activity for the DMN and all brain regions,^27-30^ aiming to identify such trigger neural zones that are crucial for the cognitive decline in Alzheimer’s disease.

INT, also known as temporal receptive windows^31-36^ or temporal receptive fields,^37^ represents the time period during which a brain region integrates neural inputs from other regions.^38^ For example, regions within the DMN and frontal–parietal network typically exhibit longer INT compared to other brain regions, which ostensibly enables the DMN to incorporate diverse neural inputs from various remote brain areas into their intrinsic signal processing.^31,32,34,36,37,39-41^ In contrast, sensory-related cortices, such as the primary visual cortex, typically exhibit a shorter INT, which permits these regions to respond to their inputs with higher temporal fidelity.^31,40,42,43^

In addition, we previously showed that this temporal index for local neural activity is related to neuroanatomical features: a brain region with a larger grey matter volume (GMV) more likely had a longer INT.^27^ This relationship between INT and GMV has biological relevance insofar as a brain area with a higher neuronal density, as proxied by a larger GMV, tends to have more recursive neural processing, which prolongs the autocorrelation of its intrinsic neural activities, thereby yielding a longer INT.

Since that GMV loss reflects atrophy in Alzheimer’s disease^44^ resulting from neuropathological processes including severe neuronal loss,^45,46^ these previous findings indicate the possibility that INT could link local neuroanatomical changes to atypical neural activity and symptomatic behaviours in neurological and neuropsychiatric disorders.^27,28^

Given this background, we focused on INT aiming to identify a key DMN region whose atrophy affected its INT, which in turn then disturbed DMN neural dynamics, which then potentially led to Alzheimer’s disease symptoms. By analysing rsfMRI data obtained from Alzheimer’s disease patients and matched healthy controls, we tested the following three hypotheses: (i) the overall INT of the entire DMN should be shortened in Alzheimer’s disease individuals and associated with Alzheimer’s disease symptoms; (ii) one or more DMN regions should also exhibit a shorter INT in the Alzheimer’s disease group, and such atypical INT in the regions should underlie the shorter INT of the entire DMN; and (iii) the DMN regions with shorter INT should have atypically smaller GMV, which should be the anatomical basis for the apparently shortened INT of the areas.

## Materials and methods

### Participants

We used an MRI dataset collected by the Knight Alzheimer Disease Research Center at Washington University in Saint Louis,^47^ which has public availability as the Open Access Series of Imaging Studies (OASIS-3).^48^ The data acquisition procedures from people diagnosed with Alzheimer’s disease and matched healthy controls received ethics approval for human subject research by the Washington University Human Research Protection Office, and all participants or their guardians provided written consent.

Participants completed clinical assessment protocols in accordance with the Uniform Data Set (UDS),^49^ which included medical history, physical examination, neuropsychological test battery and neurological evaluation. The diagnosis of Alzheimer’s disease was based on NINCDS-Alzheimer’s diseaseRDA criteria.^50^ The Clinical Dementia Rating (CDR) was also assessed as a measure of the severity of dementia:^51,52^ individuals with more than a zero CDR score were diagnosed as having Alzheimer’s disease (‘Alzheimer’s disease dementia’), while those with zero CDR score were labelled as non-demented (‘cognitively normal’, CN).^47,48,53^

Aiming to reduce within-group heterogeneity, we excluded Alzheimer’s disease individuals who showed relatively normal scores in the mini-mental state examination (MMSE), which is a widely used measure of preserved general cognitive function.^54^ We thus concentrated on Alzheimer’s disease individuals with MMSE scores ≤ 23.^55,56^

We also excluded Alzheimer’s disease participants whose clinical assessment occurred either more than one year before or after the MRI scan, whose head motion during scanning exceeded 3 mm, or who had dementia-relevant cardiovascular diseases or strokes. The control group included age-/sex-/handedness-matched adults. Specifically, the dataset initially contained 460 participants who met NINCDS-ADRDA criteria for probable Alzheimer’s disease or possible Alzheimer’s disease. Of these, 200 participants had clinical assessment within one year of their rsfMRI data. Participants were then excluded if they: (i) had excessive head motion (*n* = 1); (ii) had one or more comorbid diagnosis (*n* = 83); or (iii) had MMSE score > 23 (*n* = 84). With these criteria, we included 32 individuals with Alzheimer’s disease and 138 CN individuals (Table 1) in our analysis.

**Table 1:** Demographic data

	Cognitively normal (CN)	Alzheimer’s disease (AD)	Statistic	*P*-value
Number of participants	138	32		
Age, mean ± SD (min–max)	77.6 ± 4.8 (72–95)	78.5 ± 7.0 (66–97)	0.93	0.35
Sex, female/male	69/69	15/17	0.10	0.75
Laterality, left/right/ambidextrous	11/124/3	2/30/0	0.84	0.66
Mini-mental state examination (MMSE)	28.8	20.5	28.20	0.001

### MRI data acquisition

The rsfMRI and anatomical MRI data were collected with a 3T MRI scanner (TIM Trio, Siemens) using a 20-channel head coil. The rsfMRI data were acquired using a multi-band echo-planar imaging sequence in a single run lasting ∼6 min (repetition time = 2.2 s, echo time = 27 ms, 36 slices, interleaved, flip angle 90°, in plane resolution = 4 × 4 mm^2^, slice thickness = 4 mm); for anatomical MRI, structural images were obtained using T_1_-weighted sequence (repetition time = 2.4 s, echo time = 3.16 ms, flip angle = 8°, in plane resolution = 1 × 1 mm^2^, slice thickness = 1 mm). The participants were asked to lie quietly with their eyes open during the rsfMRI scanning.

### Preprocessing of rsfMRI data

The rsfMRI data were preprocessed with SPM12 (www.fil.ucl.ac.uk/spm) after discarding the first five volumes. This preprocessing procedure consisted of realignment, unwarping, slice-timing correction and normalization to a standard template (ICBM 152). We then performed regression analyses to remove the effects of head motion, white matter signals and cerebrospinal fluid signals, and finally conducted band-pass temporal filtering (0.01–0.1 Hz). Note that participants who had high head motion (mean motion > 3 mm) were excluded, which resulted in no significant difference in the mean head motion (*P* > 0.1 in a two-sample *t*-test) or maximum/mean frame-wise displacement (*P* > 0.2 in a two-sample *t*-test) between the Alzheimer’s disease and CN groups.

### Statistical analyses

#### Whole-brain INT analysis

**Figure 1:**
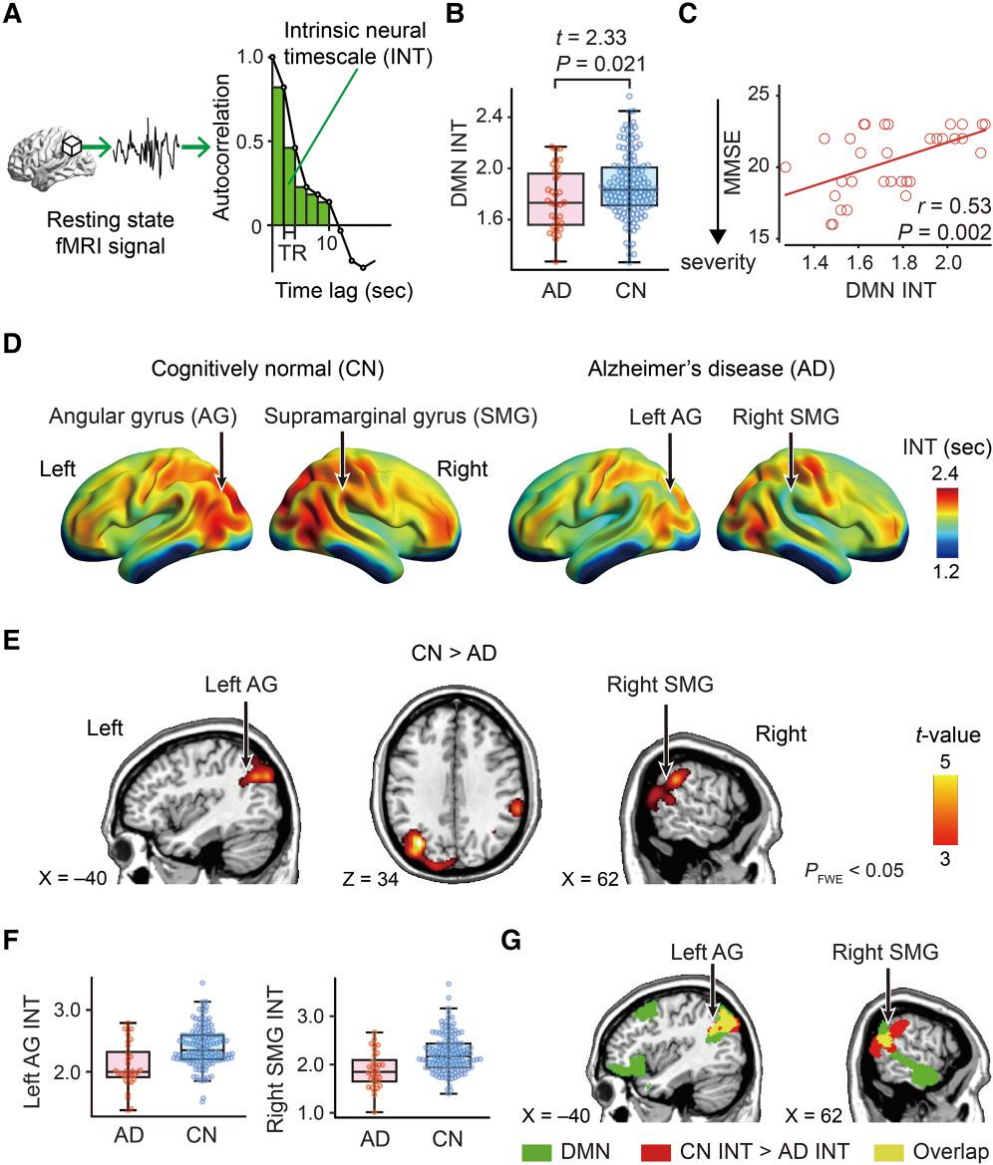
**Intrinsic neural timescale and cognitive impairment.** (**A**) To estimate an intrinsic neural timescale (INT), we calculated the area under the curve of the autocorrelation function of each local resting-state functional MRI (fMRI) signal. (**B**) INT of the default mode network (DMN) was significantly shorter in the Alzheimer’s disease group compared to the cognitively normal individuals (CN). (**C**) In the Alzheimer’s disease group, DMN INT correlated with their Alzheimer’s disease symptoms (mini-mental state examination, MMSE). Each circle represents an Alzheimer’s disease individual. (**D**) Exploratory whole-brain analyses showed longer INT in the frontoparietal areas in both the CN and Alzheimer’s disease groups. (**E** and **F**) A voxel-wise comparison of INT between the CN and Alzheimer’s disease groups showed that the Alzheimer’s disease individuals had significantly shorter INT in the left angular gyrus (AG) and the right supramarginal gyrus (SMG, *P*_FWE_ < 0.05 in a two-sample *t*-test; Table 2). No region showed a significantly longer INT in the Alzheimer’s disease group. **F** shows INT of those regions for visualization purposes. (**G**) The DMN partially overlapped with the left AG and the right SMG that showed shorter INT in the Alzheimer’s disease group relative to the CN group.

Using the preprocessed rsfMRI data, we evaluated INT for all brain regions at a single-participant level following previously used procedures.^27,28^ First, an autocorrelation function (ACF) of the preprocessed rsfMRI signal (time bin = TR) was estimated, and the area under the curve of the ACF values was calculated within the initial period during which the ACF showed positive values (Fig. 1A). The point at which the ACF first reached zero was the upper limit of the initial period. The area under the curve of the ACF was subsequently multiplied by the repetition time to define INT. After repeating this procedure for every voxel, we smoothed the resulting whole-brain INT map using a Gaussian kernel, full-width at half maximum = 8 mm. The source code for the INT analysis is available on GitHub (https://github.com/takamitsuwatanabe/IntrinsicNeuralTimescale).

#### DMN INT

Using the whole-brain INT maps, we first investigated INT within the entire DMN and tested our first hypothesis related to the association between the DMN INT and Alzheimer’s disease. The DMN was defined by a functionally-determined brain parcellation system.^57^ We calculated the DMN INT by averaging INT across all voxels within the DMN, within-subjects, for all rsfMRI time-series data and then compared INT of the DMN between the Alzheimer’s disease and CN groups. Effect sizes were reported as *η*^2^ (0.01 = small effect, 0.06 = medium effect and 0.14 = large effect).^58^ We further computed Pearson correlation coefficients between DMN INT and the severity of Alzheimer’s disease symptoms (MMSE scores). Since a Shapiro–Wilk test indicated non-normal distribution for MMSE scores (*P* = 0.002), statistical significance was tested with non-parametric bootstrapping approach (10 000 samples). For these data and other statistical tests, statistical significance was set to *P* < 0.05. We report exact probabilities greater than *P* = 0.001 and use the less-than-equal symbol for *P*-values < 0.001. We applied Bonferroni correction for multiple comparisons where appropriate.^59^

#### Explanatory whole-brain analysis

To examine our second hypothesis, we used the whole-brain INT maps and searched for the DMN regions whose INT were atypically shorter in the Alzheimer’s disease individuals. We chose the explanatory whole-brain analysis so as to exclude the possibility that brain areas outside of the DMN showed significantly shorter INT and affected the DMN activity.

We first estimated the group-mean maps by averaging them within groups to show whole-brain patterns of INT. Then, we compared INT between the Alzheimer’s disease and CN groups in a voxel-wise manner across the entire brain, based on a random-effects model using individual INT maps in both groups with age and sex information included as nuisance variables. Using family-wise error (FWE) correction at the cluster level for multiple comparisons across the whole brain, based on random field theory, we set a statistical threshold at *P*_FWE_ = 0.05.

We then examined whether the regions with shorter INT in the Alzheimer’s disease group were spatially included in the DMN area.

#### Mediation relationships between Alzheimer’s disease symptoms, DMN INT and local INT

Next, using mediation analysis, we investigated the relationships between the Alzheimer’s disease symptoms, shorter INT of the DMN and shorter INT of the focal DMN regions.

Since a mediation analysis requires significant pairwise correlations between all the three factors, we first assessed Pearson product moment correlation coefficients between the three factors except for the correlation between the DMN INT and Alzheimer’s disease symptoms, which was already previously calculated. That is, we calculated correlation coefficients between (a) the DMN INT and local INT and then (b) between the local INT and Alzheimer’s disease symptoms (MMSE scores). The local INT of the DMN regions were given as the average INT of the regions of interest (ROIs) that were defined as a 4 mm-radius sphere centred at the peak coordinates found in the above whole-brain analysis. The effects of multiple comparisons between these ROIs were corrected using the Bonferroni correction. We conducted the mediation analysis only for the ROIs whose INT met all the requisite conditions.

Note that this spatial overlap between the target ROI (here, left AG or right SMG) and the DMN does not violate any prerequisite for the mediation analysis. First, biologically, it is not always self-evident that the INT of a single DMN region would control the entire DMN INT. Statistically, mediation analysis does not require independence between the exposure, mediator and outcome variables.^60^ Rather, as a prerequisite, the three types of variables for mediation analysis should be significantly correlated with each other.

We performed two types of mediation analyses to test the following two models: (a) the atypically shorter local INT shortens INT of the DMN, which results in the Alzheimer’s disease symptoms; and (b) the atypicality of the local INT is strong enough to account for the link between DMN INT and Alzheimer’s disease symptoms.

To test model (a), we set the local INT as an independent variable and defined the mediator variable using DMN INT. To test model (b), we used DMN INT as an independent variable and adopted the local INT as a mediator variable. In both cases, the MMSE scores indicating the Alzheimer’s disease symptoms were set as a dependent variable. The statistical significance of the mediation effect was tested using non-parametric bootstrap procedure with 10 000 iterations.

After determining which model worked in the current dataset, we examined the specificity of the DMN in this brain–behaviour relationship. For this control test, we conducted another mediation analysis after replacing DMN INT with INT of the other six brain networks defined in the same brain parcellation system,^57^ i.e. frontal–parietal network, dorsal attention network, ventral attention network, limbic system, sensory-motor network and visual network.

#### GMV of the focal DMN regions

To test our third hypothesis, we calculated the GMV of the ROIs that were detected in the previously described analysis. We estimated the GMV based on the T_1_-weighted MRI images using SPM12 for each participant as follows. First, the MRI images were segmented into grey matter, white matter and cerebrospinal fluid with the New Segment Toolbox.^61^ Second, using the DARTEL Toolbox,^62^ the segmented grey matter images were aligned, warped to a template space, resampled to 1.5-mm isotropic voxels. The grey matter images were then smoothed with a Gaussian kernel (full-width at half maximum = 8 mm). Using these preprocessed T_1_-weighted images, we then calculated the GMV of the ROI that was detected in the above analysis.

#### Mediation relationships between the local GMV, local INT and DMN INT

We then examined mediation relationships between the local GMV, local INT and DMN INT using mediation analysis methods. First, in preparation for the analysis, we calculated the Pearson product moment correlation coefficients among the three factors. In practice, we assessed correlation coefficients (a) between the local GMV and local INT and then (b) between the local GMV and DMN INT, because the pairwise correlation between the local INT and DMN INT was already confirmed in the above analysis.

We then conducted a non-parametric mediation analysis with the local GMV as an independent variable, local INT as a mediator variable and DMN INT as a dependent variable. The statistical significance of the mediation effect was calculated using a bootstrapping method with 10 000 iterations.

As a control, we also conducted the same mediation analysis using the GMV and INT of the brain regions in the contralateral hemisphere compared to the original ROIs that were identified in the whole-brain exploratory analysis and whose INT correlated with Alzheimer’s disease symptoms.

To confirm whether the local GMV affected the Alzheimer’s disease symptoms via the local INT, we used the original ROIs and performed a mediation analysis with the local GMV as an independent variable, local INT as a mediator variable and MMSE scores as a dependent variable.

#### Associations with different cognitive components

Finally, we searched for cognitive components that were specifically affected by the changes in DMN INT. To this end, we conducted a regression analysis between DMN INT and the scores of the following neuropsychological tests^49,63^: ‘Digit Span Test’ for the measurement of attention; ‘Trail Making Test Part A’ and ‘Digit Symbol Test’ to evaluate processing speed; ‘Trail Making Test Part B’ to quantify executive function; ‘Logical Memory Test’ to measure episodic memory; and ‘Boston Naming Test’ and ‘Category Fluency Test’ to evaluate language function. All the scores were converted to *Z*-scores. The scores of Trail Making Tests were inverted to represent that a higher value indicated better cognitive performance.

## Results

### INT of DMN

To test our first hypothesis, we investigated INT (Fig. 1A) of the DMN and found that DMN INT was significantly shorter in the Alzheimer’s disease individuals than in the cognitively normal (CN) group (*t*_168_ = 2.33, *P* = 0.02, *η*^2^ = 0.03 in a two-sample *t*-test; Fig. 1B). The atypically shorter DMN INT significantly correlated with the severity of Alzheimer’s disease (*r* = 0.53, *P* < 0.001; Fig. 1C). These results support our first hypothesis that the overall INT of the entire DMN should be shortened in Alzheimer’s disease individuals and associated with Alzheimer’s disease symptoms.

### Whole-brain analysis of INT

Next, to test our second hypothesis, we performed an exploratory whole-brain analysis of INT (Fig. 1D) and found that the Alzheimer’s disease group exhibited significantly shorter INT compared to the CN group, but only in the left angular gyrus (AG) and right supramarginal gyrus (SMG) (*P* < 0.001, *P*_FWE_ < 0.001 for the left AG, *P* = 0.002, *P*_FWE_ = 0.007 for the right SMG; Fig. 1E and F, and Table 2). No region showed a significantly longer INT in the Alzheimer’s disease individuals compared to the controls. Both the AG and SMG are included within the DMN (Fig. 1G).

**Table 2:** Results of whole-brain analysis of intrinsic neural timescale

	Region	Laterality	MNI coordinates			Cluster size (voxels)	*t*-Value
			*x*	*y*	*z*		
CN > Alzheimer’s disease	Angular gyrus	Left	−34	−70	34	3466	5.15
	Supramarginal gyrus	Right	60	−38	30	1383	4.38

### Mediation relationships between local INT, DMN INT and Alzheimer’s disease symptoms

To understand more about potential interactions within the DMN, we next evaluated the mediation relationships between the local INT, DMN INT and Alzheimer’s disease symptoms using a non-parametric mediation analysis.

**Figure 2:**
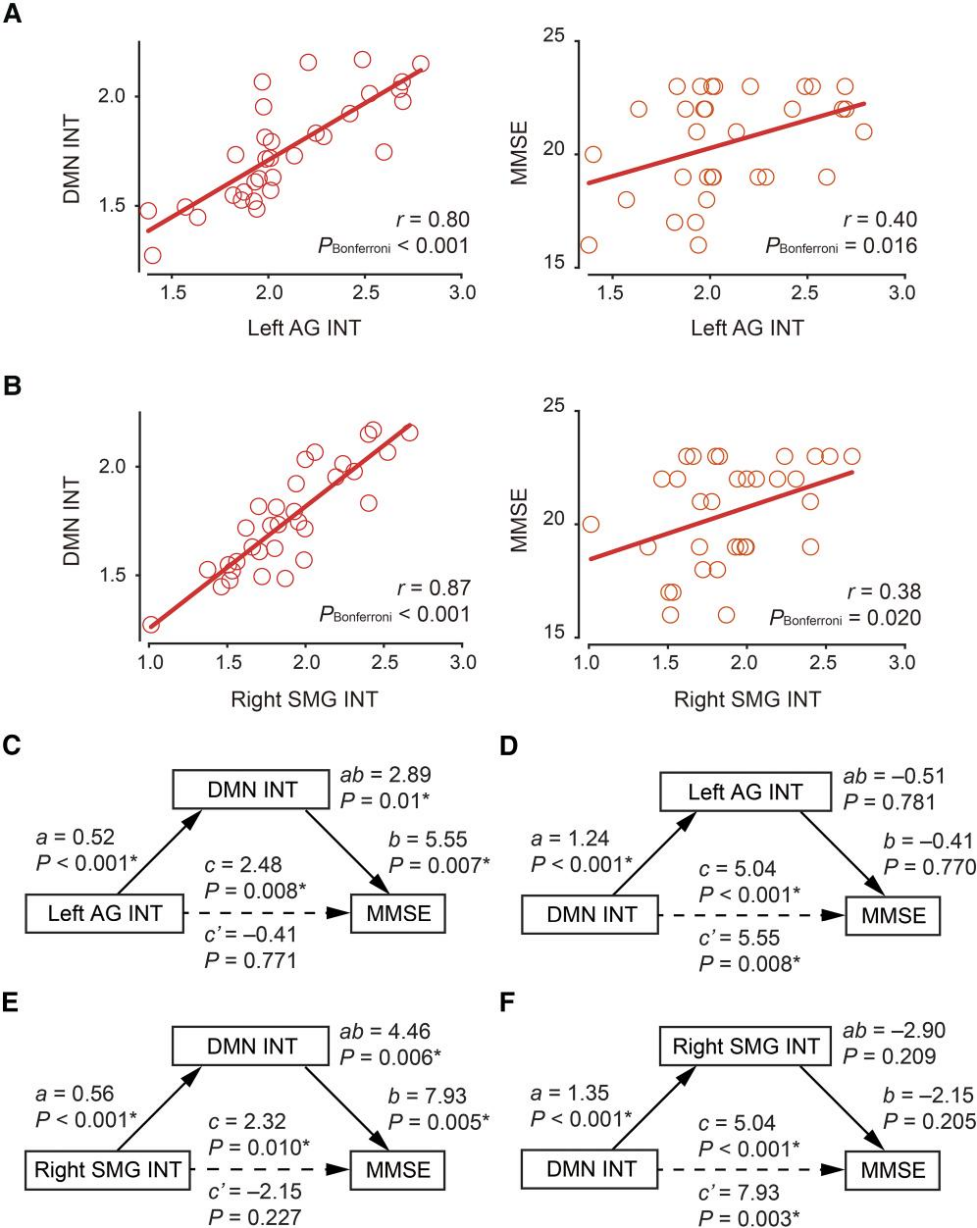
**Links between intrinsic neural timescale of local brain regions and default mode network and cognitive impairment.** (**A**) The intrinsic neural timescale (INT) of the left angular gyrus (AG) was correlated with INT of the default mode network (DMN) and symptoms of Alzheimer’s disease. The symptoms were assessed by mini-mental state examination (MMSE). Each circle represents an individual. (**B**) In the Alzheimer’s disease individuals, INT of the supramarginal gyrus (SMG) was correlated with DMN INT and MMSE. (**C**) In the Alzheimer’s disease group, a non-parametric mediation analysis showed that DMN INT accounts for the influence of AG INT on cognitive impairments of Alzheimer’s disease. *a* denotes a regression coefficient of AG INT on DMN INT, and *b* indicates that of DMN INT on MMSE. *c* represents the direct effect of AG INT on MMSE without the mediator effect, whereas *c*′ represents that with the mediator effect of DMN INT. *ab* indicates the indirect effect of AG INT on MMSE via DMN INT. Asterisk indicates *P*_Bonferroni_ < 0.05 between the two ROIs. (**D**) A separate mediation analysis suggests that AG INT cannot explain the association between DMN INT and Alzheimer’s disease symptoms. (**E**) A mediation analysis suggests that DMN INT explain the association between SMG INT and Alzheimer’s disease symptoms. (**F**) A mediation analysis suggests that SMG INT cannot explain the association between DMN INT and Alzheimer’s disease symptoms.

First, we examined whether the three factors, that is, local INT, DMN INT and Alzheimer’s disease symptoms, were significantly correlated with each other, since mediation analysis requires such pairwise correlations. In the case of the left AG, we satisfied the requisite conditions; left AG INT and DMN INT: *r* = 0.80, *P*_uncorrected_ < 0.001, *P*_Bonferroni_ < 0.001; left AG INT and MMSE: *r* = 0.40, *P*_uncorrected_ = 0.008, *P*_Bonferroni_ = 0.016; Fig. 2A. These preconditions for the right SMG were also met; SMG INT and DMN INT: *r* = 0.87, *P*_uncorrected_ < 0.001, *P*_Bonferroni_ < 0.001; that between right SMG INT and MMSE: *r* = 0.38, *P*_uncorrected_ = 0.01, *P*_Bonferroni_ = 0.02; Fig. 2B. Given these results, we focused on the left AG and the right SMG for all subsequent analyses.

In the mediation analysis, we tested the following two models to understand the interrelationships between the left AG INT, DMN INT and Alzheimer’s disease symptoms (MMSE score): first, we assessed whether atypically shorter left AG INT reduces DMN INT and consequently contributes to Alzheimer’s disease symptoms, and second, we examined whether the shortened left AG INT can explain the association between DMN INT and Alzheimer’s disease symptoms.

The outcome of the mediation analyses supported the first model. That is, DMN INT mediated the entire relationship between left AG INT and Alzheimer’s disease symptoms (*P* = 0.01, *P*_Bonferroni_ = 0.02; Fig. 2C). By contrast, left AG INT did not account for the significant direct association between reduced DMN INT of Alzheimer’s disease patients and their symptoms (*P* = 0.78, *P*_Bonferroni_ > 0.99; Fig. 2D).

We next conducted the mediation analysis with respect to the right SMG in the same way as the left AG. The outcome supported the mediation model in which the shortened right SMG INT reduced DMN INT and consequently contributes to Alzheimer’s disease symptoms (*P* = 0.006, *P*_Bonferroni_ = 0.012; Fig. 2E). Meanwhile, right SMG INT did not explain the direct effect of DMN INT on Alzheimer’s disease symptoms (*P* = 0.21, *P*_Bonferroni_ = 0.42; Fig. 2F).

To control for the possibility that reduced DMN INT simply reflected a generalized decrease in neural dynamics across all brain regions, we checked differences in INT of brain networks between the Alzheimer’s disease and CN groups (Supplementary Table 1) and then assessed whether the INT of brain networks other than the DMN mediated the direct effect of left AG INT on the MMSE score. Regarding the left AG, we found that, except for the DMN, none of the brain networks exhibited atypical INT that could explain the direct influence of the left AG INT on the MMSE score (mediation effects, frontoparietal network: *P* = 0.11; dorsal attention network: *P* = 0.09; ventral attention network: *P* = 0.19; limbic system: *P* = 0.59; sensory-motor network: *P* = 0.20; visual network: *P* = 0.88). Likewise, none of the six brain networks exhibited any significant mediation effect on the relationship between the right SMG INT and the MMSE score (mediation effects, frontoparietal network: *P* = 0.10; dorsal attention network: *P* = 0.10; ventral attention network: *P* = 0. 23; limbic system: *P* = 0.72; sensory-motor network: *P* = 0.31; visual network: *P* = 0.84). That is, even though the right SMG spatially overlapped with the DMN, frontoparietal network and ventral attention network, only the DMN exhibited the mediation effect on the relationship between the local INT and Alzheimer’s disease symptoms. These null results provide further evidence that the DMN has a specific functional role in bridging the gap between atypical neural processing in the local brain regions and cognitive impairment of Alzheimer’s disease.

### GMV of AG and SMG

**Figure 3:**
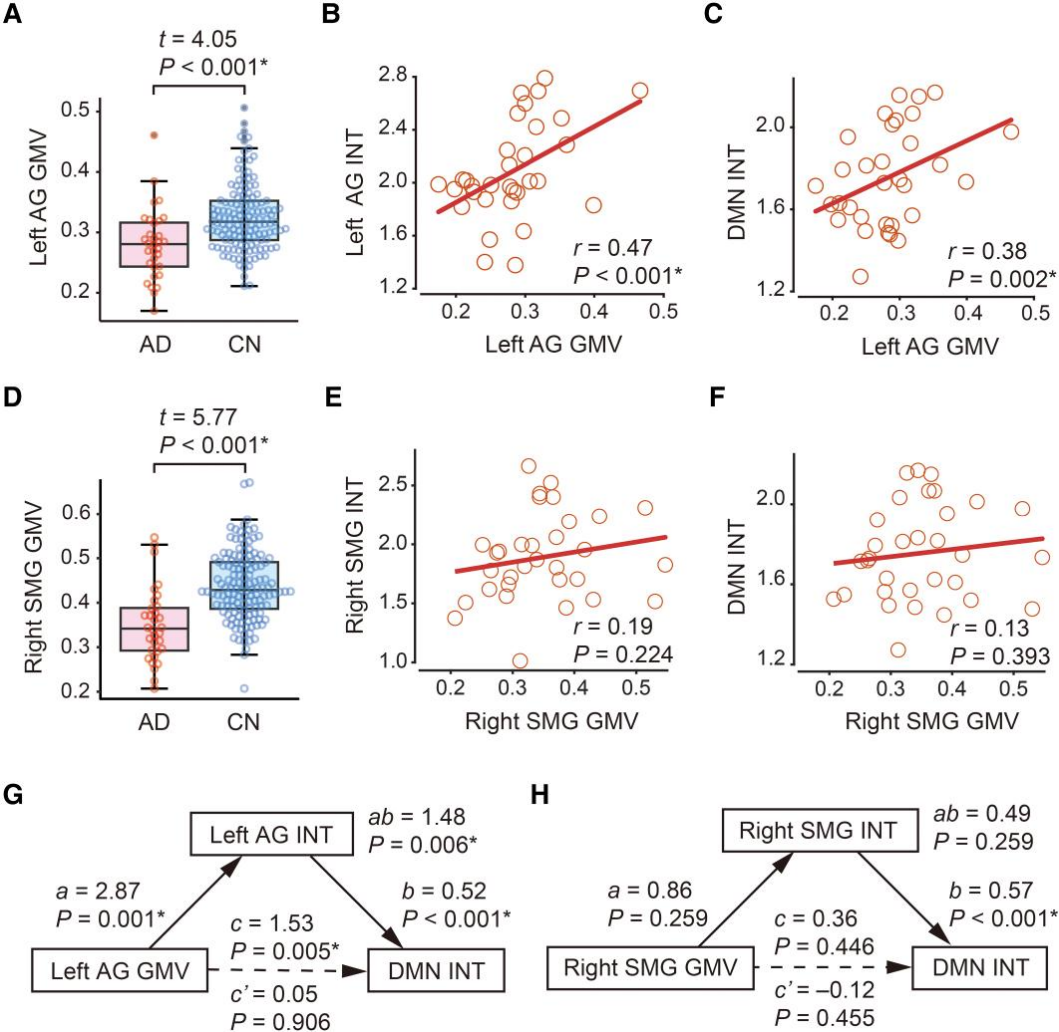
**Association between grey matter volume and intrinsic neural timescale.** (**A**) The grey matter volume (GMV) of the left angular gyrus (AG) was smaller in the individuals with Alzheimer’s disease and cognitively normal (CN) group. (**B**) In the Alzheimer’s disease group, AG GMV was significantly correlated with the intrinsic neural timescale (INT) of the left AG. Each circle represents an individual. (**C**) In the Alzheimer’s disease individuals, AG GMV was also correlated with INT of the default mode network (DMN). (**D**) The GMV of the right supramarginal gyrus (SMG) was smaller in the Alzheimer’s disease group than in the CN group. (**E**) In the Alzheimer’s disease group, SMG GMV was not significantly correlated with SMG INT. (**F**) In the Alzheimer’s disease individuals, SMG GMV was not correlated with DMN INT. (**G**) A non-parametric mediation analysis showed the indirect effect of the atypically reduced AG GMV on shorter DMN INT that is mediated by shortened AG INT. (**H**) We did not find such a significant mediation effect for the right SMG. Asterisk indicates *P*_Bonferroni_ < 0.05 between the two regions of interest.

To test our third hypothesis that the DMN regions with shorter INT would have atypically smaller GMV, we calculated the GMV of the left AG and right SMG and confirmed that GMV in both the AG and SMG was significantly reduced in the Alzheimer’s disease group compared to that in the CN group (left AG: *t*_168_ = 4.05, *P* ≤ 0.001, *P*_Bonferroni_ ≤ 0.001, *η*^2^ = 0.09, Fig. 3A; right SMG: *t*_168_ = 5.77, *P* ≤ 0.001, *P*_Bonferroni_ ≤ 0.001, *η*^2^ = 0.17, Fig. 3D in a two-sample *t*-test). Additionally, GMV in the left AG correlated significantly with left AG INT in the Alzheimer’s disease group (*r* = 0.47, *P* = 0.006, *P*_Bonferroni_ = 0.012; Fig. 3B). This GMV–INT association has consistency with our previous finding.^27^ However, GMV in the right SMG did not correlate with right SMG INT in the Alzheimer’s disease group (*r* = 0.19, *P* = 0.22, *P*_Bonferroni_ > 0.99). Given these results, we then focused on the left AG in subsequent mediation analyses.

### Mediation relationships between AG GMV, AG INT and DMN INT

Next, we investigated the mediation relationships between the left AG GMV, left AG INT and DMN INT in the Alzheimer’s disease individuals using the non-parametric mediation analysis. All the requisite conditions for the analysis (i.e. significant Pearson product moment correlations between the three factors) were met (Fig. 3B for the correlation between AG GMV and AG INT; Fig. 2A for that between AG INT and DMN INT; Fig. 3C for that between AG GMV and DMN INT).

The mediation analysis revealed that the total effect of left AG GMV on DMN INT (*c* = 1.53, *P* = 0.005; Fig. 3G) was entirely explained by a mediation effect of left AG INT (*c*′ = 0.05, *P* = 0.91; *ab* = 1.48, *P* = 0.006). That is, this complete mediation model suggests that the reduced GMV of the left AG diminishes left AG INT, which then causes the shorter DMN INT.

To assess the specificity of the left AG GMV on DMN neural dynamics, we assessed whether GMV and/or INT of the other DMN regions mediated the total DMN INT. Since INT in the right SMG differed between the Alzheimer’s disease and CN groups (Fig. 1F) and showed a significant correlation with cognitive decline in the Alzheimer’s disease individuals (Fig. 2B), we reasoned that SMG INT might also exert a mediation effect on the relationship between SMG GMV and DMN INT. However, the requisite conditions for the mediation analysis were not met (Fig. 3E and F), and the analysis failed to show a significant mediation effect (*P* = 0.26; Fig. 3H). Moreover, we confirmed that INT of the right AG or left SMG, other DMN regions and counterpart of the left AG or right SMG, did not have a significant mediation effect on the relationship between GMV of the right AG or left SMG and DMN INT (right AG: *P* = 0.76; left SMG: *P* = 0.44, Supplementary Fig. 1). We also found no significant mediation effect for any of the other DMN regions (superior medial prefrontal gyrus, anterior cingulate, bilateral superior frontal gyri, bilateral inferior temporal gyri, bilateral parahippocampal gyri and middle cingulate). These negative findings suggest that the left AG has a specific role in linking its local anatomical change to the overall DMN activity in Alzheimer’s disease individuals.

We further evaluated whether the left AG GMV affected MMSE scores via left AG INT by confirming that the mediation effect of the left AG INT was not significant (*P* = 0.17, Supplementary Fig. 2). This result strengthens our observation of the indirect effect of left AG INT on MMSE scores via the DMN INT (Fig. 2C).

**Figure 4:**
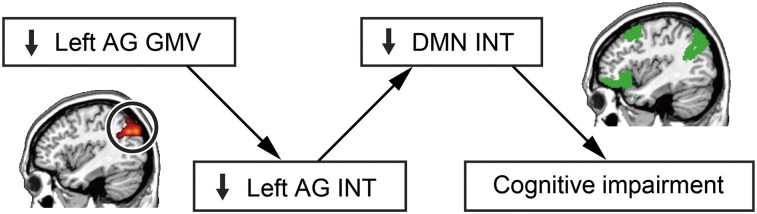
**Links between neuroanatomical, functional and behavioural changes in Alzheimer’s disease.** The two mediation analyses (Figs 2C and 3G) suggest that the atypically smaller grey matter volume (GMV) of the left angular gyrus (AG) shortened its intrinsic neural timescale (INT), and such shorter AG INT reduced INT of the default mode network (DMN), which led to the cognitive impairment of Alzheimer’s disease. The left AG and DMN were shown schematically based on Fig. 1E and G.

Considering this result (Fig. 3G) along with other findings (Fig. 2C), we inferred that, in the Alzheimer’s disease patients, their atypical reduction in GMV of the left AG shortened its INT, shorter AG INT reduced DMN INT, and finally that the shorter DMN INT led to their cognitive impairment (Fig. 4).

### Specific cognitive components

**Figure 5:**
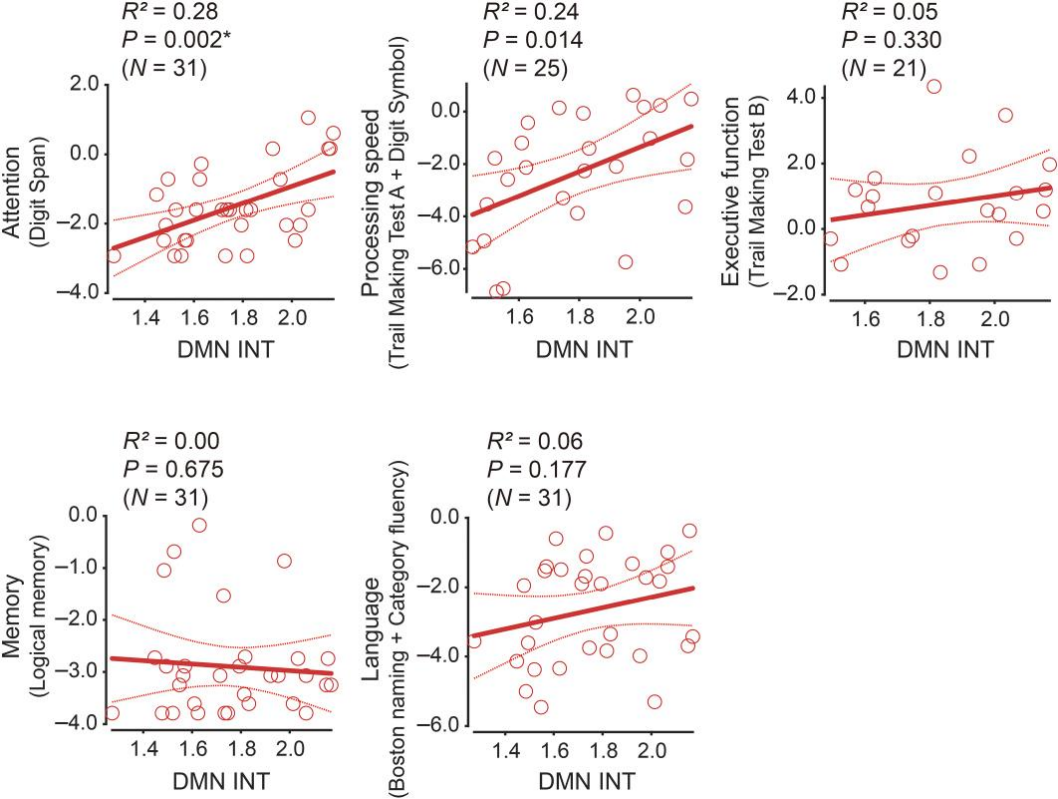
**Associations between intrinsic neural timescale of default mode network and different cognitive components.** In the group with Alzheimer’s disease, the intrinsic neural timescale (INT) of the default mode network (DMN) was significantly associated with attention but neither with processing speed, executive function, memory nor language. Each circle represents an individual. Asterisk indicates *P*_Bonferroni_ < 0.05 between the five cognitive components.

Finally, we assessed whether DMN INT exerted specific effects on cognitive components in Alzheimer’s disease (Fig. 5). We found that DMN INT correlated significantly in the Alzheimer’s disease patients only with attention (*r* = 0.47, *P* = 0.007, *P*_Bonferroni_ = 0.035) but not with processing speed (*P* = 0.014, *P*_Bonferroni_ = 0.07), executive function (*P* = 0.33, *P*_Bonferroni_ > 0.99), memory (*P* = 0.68, *P*_Bonferroni_ > 0.99) or language (*P* = 0.18, *P*_Bonferroni_ = 0.89). These results suggest an association between the alteration of DMN INT in Alzheimer’s disease and changes in attention systems. Note that this correlation analysis was based on different sample sizes since some behavioural scores did not have availability for some patients in the dataset we used.

## Discussion

By focusing on INT, we provided a model in which the left AG as a key region whose atrophy induces its atypical intrinsic neural activity, which then disturbs neural dynamics of the entire DMN and consequently leads to cognitive impairment in Alzheimer’s disease, especially attention-related symptoms. Given that INT is related to the capacity of local information integration,^40,42,64-66^ these findings suggest that, in individuals with Alzheimer’s disease, the atrophy of the left AG affects the capability of the DMN to integrate a wide range of neural inputs to the network, which then appears to diminish efficient cognitive processing and results in some of the Alzheimer’s disease symptoms, such as impaired attention control.

The ostensible linkage between shorter DMN INT and Alzheimer’s disease symptoms is consistent with previous studies on the functional roles of the DMN in healthy humans. The DMN is known to have a central role in orchestrating diverse cognitive functions in older individuals.^25^In the meantime, several human neuroimaging studies reported that DMN regions are likely to exhibit longer INT than those in sensory-related brain networks,^30,64,65,67^ and such longer INT of the DMN is considered to allow of the network to pool and integrate neural inputs from other regions for complex behavioural and cognitive processes.^40,42,64-66^ Given these previous reports, the current findings on the associations between the atypically shorter DMN INT and Alzheimer’s disease symptoms appear to be neurobiologically reasonable.

In addition to the DMN INT, we examined INT of the other attention-related brain networks—i.e. the dorsal attention network (DAN) and ventral attention network (VAN)—and explored the relationship between their INT and cognitive performance. As a result, we found a significant decrease in INT of the DAN but did not for the VAN. In addition, the DAN INT was correlated with the performance in attention control. These observations imply that attention-related symptoms in Alzheimer’s disease may not be solely associated with the DMN but might also involve the DAN.

Both the left AG and right SMG exhibited atypical INT and their INT were similar to each other (*r* = 0.74, *P* < 0.001). In fact, their shortened INT was linked with cognitive impairment via the DMN INT (Fig. 2C and E). However, it was only the left AG INT that had a mediation effect on the relationship between its GMV and DMN INT. Given these findings, although these regions showed similar neural dynamics, the left AG seems to be more plausible as a source of atypical INT than the right SMG.

The microscopic association between the INT and neuronal density remains uncertain. The significant correlation observed between the left AG INT and its GMV provides face validation for our hypothesis that a decreased local neuron number in Alzheimer’s disease would reduce the autocorrelation of its intrinsic neural activities and shorten INT. Also, this finding is consistent with previous reports on a significant correlation between grey matter atrophy and neuronal loss in Alzheimer’s disease.^45,46^ However, other studies reported that atrophy did not reflect neuronal density change.^68,69^ Given these, future studies focusing on biological microarchitectures would be necessary to reveal more precise associations between the INT, GMV and atrophy.

The crucial role of the AG in Alzheimer’s disease has been supported by previous findings. Several rsfMRI studies observed that the AG showed atypically weak functional connectivity between the DMN and other brain regions.^70,71^ Research employing positron emission tomography reported hypometabolism, atrophy and amyloid and tau deposition in the parietal regions—including the AG—in individuals with Alzheimer’s disease.^26,72-75^ The current observation about the importance of the left AG in Alzheimer’s disease-related cognitive decline would fill a gap between AG anatomy and function and provide a more integrated view on the biological mechanisms underpinning Alzheimer’s disease.

Our findings suggest the possibility that the left AG could be a target area for non-invasive brain stimulation to slow or even modify Alzheimer’s disease symptoms. Indeed, accumulating evidence shows the beneficial effects of repetitive transcranial magnetic stimulation (rTMS) on Alzheimer’s disease^76^: for example, a recent rTMS study demonstrated that stimulation of the precuneus, which is a part of the DMN, could mitigate the cognitive decline seen in Alzheimer’s disease patients.^77^ Given the known involvement of the DMN neural dynamics in various cognitive processes that are often impaired in Alzheimer’s disease, an effective approach to mitigate the DMN dysfunction is desirable. Considering our findings, a brain stimulation method to modify INT of the AG could potentially modulate the atypical DMN brain dynamics and alleviate the progressive cognitive impairments of the prevalent neurodegenerative dementia.

To reduce the heterogeneity within the Alzheimer’s disease group, we applied stringent selection criteria, which consequently decreased the sample size of this study. Despite this, a power analysis with the current sample size, a significance level of *α* = 0.05 and effect size (Cohen’s d) based on data of our previous study^27^ indicated that the current study has a high statistical power (1 − *β* = 0.96). In addition, when we adopted relaxed selection criteria to select Alzheimer’s disease patients (MMSE score ≤ 24–26), we observed that all the significant INT differences in the whole-brain analysis disappeared along with the relaxing of the selection threshold. This outcome using relaxed criteria suggests that the strict initial selection criteria may minimized within-group heterogeneity and enabled us to identify Alzheimer’s disease-specific neural dynamics.

Our findings, therefore, cannot be immediately generalized to more heterogeneous Alzheimer’s disease cases, since the current examination excluded MCI patients and the individuals with the early stage of Alzheimer’s disease. Although a ROI-based analysis showed a significant shorter INT in the left AG with a relaxed selection threshold for Alzheimer’s disease (MMSE ≤ 26; *t*_218_ = 3.03, *P* = 0.003, *η*^2^ = 0.04 in a two-sample *t*-test), it would be still necessary to conduct future research employing a larger and more diverse dataset to test whether INT of the left AG can serve as a biomarker for Alzheimer’s disease. If the current finding were verified in such future studies, a mere 15-min structural and functional MRI scan would enable a facile evaluation of the degree of the neurodegeneration and resultant dysfunction of the DMN in individuals with Alzheimer’s disease.^78^

One limitation of the current study is that we used a cross-sectional dataset with a relatively limited Alzheimer’s disease sample size. Nevertheless, the mediation analyses direct links between local atrophy, local brain dynamics, network neural dynamics and behaviour (Fig. 4). Furthermore, to validate the mediation model in which the smaller AG GMV would shorten AG INT and subsequently decrease DMN INT, we examined an alternative mediation model that assumed that the shorter AG INT should reduce AG GMV and decrease DMN INT. This sensitivity analysis yielded a null result (Supplementary Fig. 3), providing additional support for the original model. However, we do note that the current study did not adopt a causal mediation analysis mainly because the data used here were not longitudinal. Instead, we used a conventional mediation analysis and, thus, longitudinal studies or intervention research will be necessary for the direct examination of the causal effects of such alteration of the local brain activity on Alzheimer’s disease symptoms.

Limited spatial resolution may cause partial volume effects at the boundaries between grey matter, white matter and CSF tissues in populations with neurodegenerative disease. Theoretically, such partial volume effects should be reduced in the current rsfMRI preprocessing that includes the exclusion of the signals of white matter and CSF, but future studies would be necessary to directly assess the partial volume effects on the accuracy and reliability of the INT calculation, especially, in neurodegenerative cohorts.

Another limitation of our study concerns the absence of directly investigating pathological links between INT and amyloid and tau depositions, which was far beyond the scope of the employed dataset. However, we confirmed the effects of the GMV reduction on the shorter INT in the left AG, and such a decrease in GMV is thought to reflect neuronal atrophy.^44^ Future studies would be necessary to directly examine the relationships between INT and amyloid/tau depositions in individuals with Alzheimer’s disease.

## Supplementary Material

fcae199_Supplementary_Data

## Data Availability

The data that support the findings of this study are from the OASIS website (https://www.oasis-brains.org/).
