# Supplementary material for: Atypical intrinsic neural timescale in the left angular gyrus in Alzheimer’s disease
Source: Brain Commun. 2024 Jul 11;6(4):fcae199. doi: 10.1093/braincomms/fcae199 (PMC11227993; doi:10.1093/braincomms/fcae199)
Supplement: fcae199_Supplementary_Data [file fcae199_supplementary_data.docx]

## **Supplementary material**

### **Supplementary Results**

**Supplementary Table 1 Group differences in INT of brain network**

| Network | *t* value | *η*^2^ | *P* value |
| --- | --- | --- | --- |
| Dorsal attention network | 2.13 | 0.03 | 0.034 |
| Default mode network | 2.33 | 0.03 | 0.021 |
| Frontoparietal network | 2.42 | 0.03 | 0.016 |
| Limbic system | 1.85 | 0.02 | 0.066 |
| Sensory-motor network | 1.06 | 0.01 | 0.293 |
| Ventral attention network | 1.71 | 0.02 | 0.089 |
| Visual network | 1.83 | 0.02 | 0.069 |


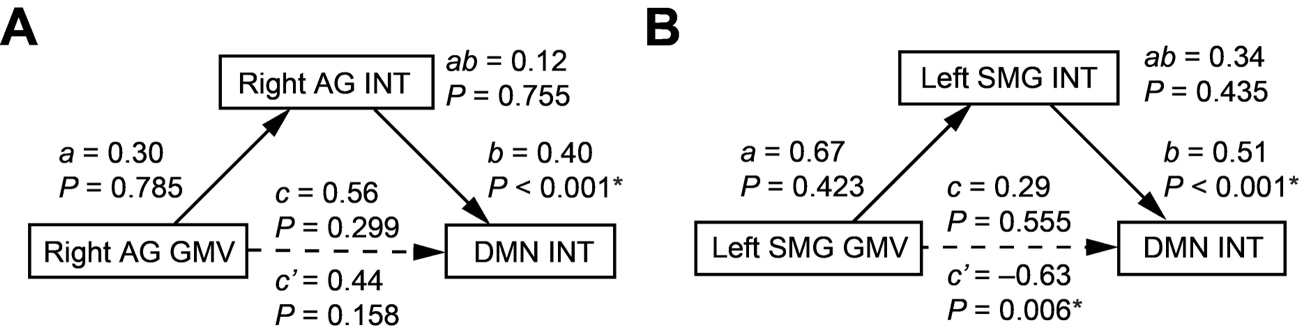


**Supplementary Figure 1. Association between grey matter volume and intrinsic neural timescale.** A mediation model in which variables of INT and GMV of the left AG or right SMG in the original model (Fig. 3G and 3H) were replaced with those of the right AG and left SMG did not result in a significant mediation effect.


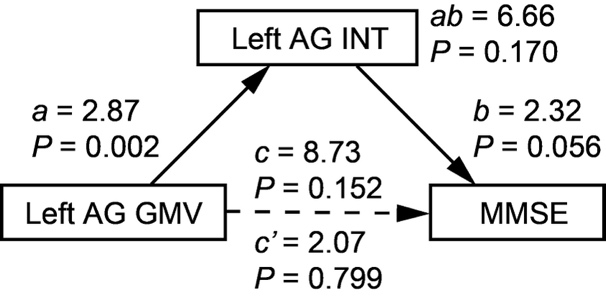


**Supplementary Figure 2. Association between grey matter volume, intrinsic neural timescale and cognitive impairment.** A mediation analysis suggests that AG INT cannot explain the association between AG INT and AD symptoms.


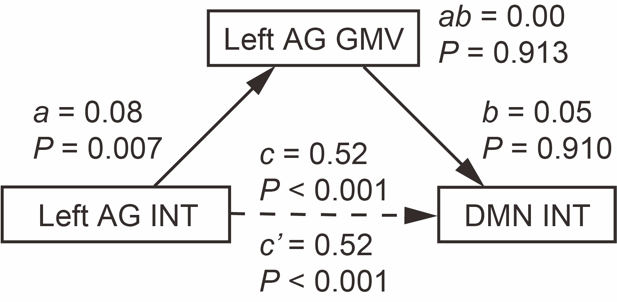


**Supplementary Figure 3. Association between grey matter volume and intrinsic neural timescale.** A mediation model in which variables of INT and GMV of the left AG were replaced in the original model (Fig. 3G) did not result in a significant mediation effect.
